# An Asymmetric Post-Hydrolysis State of the ABC Transporter ATPase Dimer

**DOI:** 10.1371/journal.pone.0059854

**Published:** 2013-04-03

**Authors:** Anthony M. George, Peter M. Jones

**Affiliations:** School of Medical & Molecular Biosciences, University of Technology Sydney, Broadway, New South Wales, Australia; Oak Ridge National Laboratory, United States of America

## Abstract

ABC transporters are a superfamily of enzyme pumps that hydrolyse ATP in exchange for translocation of substrates across cellular membranes. Architecturally, ABC transporters are a dimer of transmembrane domains coupled to a dimer of nucleotide binding domains (NBDs): the NBD dimer contains two ATP-binding sites at the intersubunit interface. A current controversy is whether the protomers of the NBD dimer separate during ATP hydrolysis cycling, or remain in constant contact. In order to investigate the ABC ATPase catalytic mechanism, MD simulations using the recent structure of the ADP+Pi-bound MJ0796 isolated NBD dimer were performed. In three independent simulations of the ADP+Pi/apo state, comprising a total of >0.5 µs, significant opening of the apo (empty) active site was observed; occurring by way of intrasubunit rotations between the core and helical subdomains within both NBD monomers. In contrast, in three equivalent simulations of the ATP/apo state, the NBD dimer remained close to the crystal structure, and no opening of either active site occurred. The results thus showed allosteric coupling between the active sites, mediated by intrasubunit conformational changes. Opening of the apo site is exquisitely tuned to the nature of the ligand, and thus to the stage of the reaction cycle, in the opposite site. In addition to this, in also showing how one active site can open, sufficient to bind nucleotide, while the opposite site remains occluded and bound to the hydrolysis products ADP+Pi, the results are consistent with a Constant Contact Model. Conversely, they show how there may be no requirement for the NBD protomers to separate to complete the catalytic cycle.

## Introduction

ABC transporters comprise the major superfamily of primary active membrane translocases. They are found in all kingdoms of life, and are engaged in multifarious cellular processes [1]–[3]. A subgroup of ABC exporters mediate resistance to structurally diverse chemotherapeutic drugs; a phenomenon known as multidrug resistance (MDR) [4]–[6]. Other ABC transporters are involved in serious and prevalent genetic diseases in humans, such as cystic fibrosis [7], [8]. In bacteria, ABC importers are essential for cell survival through their role in nutrient uptake and osmoregulation, while in pathogenic microbes and parasites, ABC exporters are involved in virulence [9]. An understanding of the molecular mechanism of ABC transporters is critical to the development of treatments for a wide range of pathological conditions affecting not only human health, but also productivity in agriculture.

A conserved core architecture of ABC transporters consists of two transmembrane domains (TMDs) and two nucleotide binding domains (NBDs). The two transmembrane domains together form a translocation channel and substrate binding sites, while the NBDs, which are located in the cytosol, bind and hydrolyse ATP [2], [10], [11]. In contrast to the TMDs, the NBDs are highly conserved, and contain a number of characteristic sequence motifs, including the Walker A and B consensus motifs for ATP binding, the LSGGQ signature sequence or C-motif, which is diagnostic of the ABC superfamily, as well as the D-, Q-, and H-loops, which are named according to conserved residues [12]–[14].

The NBD has a bilobal structure, consisting of a nucleotide binding core subdomain, and a helical subdomain, which includes the ABC signature sequence C-motif and is unique to the ABC ATPase fold. A feature of emerging importance to the functioning of the NBD is the flexible nature of the connection between the core and helical subdomains. This was inferred from comparative crystallographic analysis [15] and also observed in MD simulations [12], [16]–[18]. Elastic network analysis of the ABC NBD monomer and dimer also revealed that relative motions between the core and helical subdomains comprise the principal dynamic modes of the subunit. This indicates such motions are intrinsic and fundamental to the contact topology of the subunit, consistent a functional role [17], [18]. More recently, experiments using the maltose permease provided biophysical evidence that significant conformational changes between the helical and core subdomains occur during the catalytic cycle [19].

**Figure 1:**
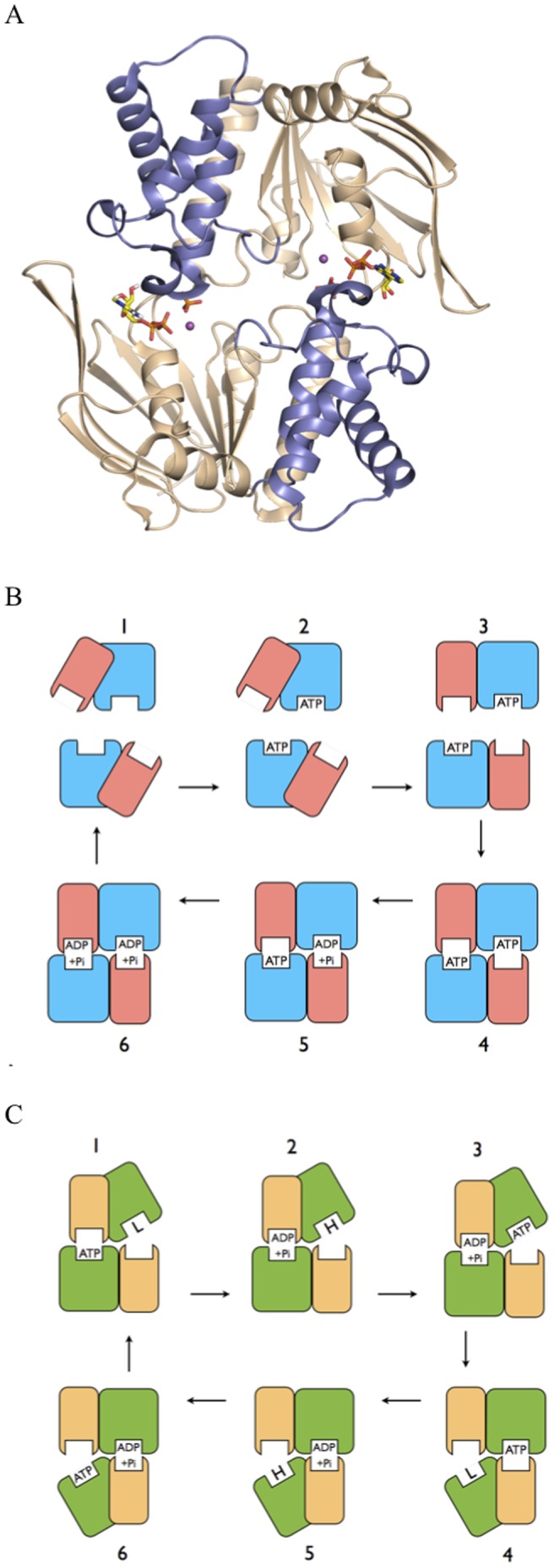
The ABC NBD Dimer. (A) NBD dimer structure. Structural figure prepared from PDB entry 3TIF, used as the starting structure in the simulations. Cartoon representation with core subdomains coloured “wheat” and helical subdomains “slate”; separate monomers are situated top and bottom. ADP+Pi molecules are bound at the intermonomer interface, and shown in stick representation, with carbon yellow, oxygen red, nitrogen blue, and phosphorous orange. Catalytic metal is shown as a purple sphere. (B) ABC Switch Model. Schematic representation of the Switch Model for the catalytic cycle of ABC transporters. Separate NBD monomers are situated top and bottom in each frame, with the larger core subdomains coloured blue and the smaller helical subdomains pink. (1) Resting state. NBDs are separated with helical domains outwardly rotated. (2) ATP binds to each NBD. (3) ATP binding induces inward rotation of helical subdomains. (4) NBDs move together forming a sandwich dimer with two occluded ATPs. (5–6) ATPs are hydrolysed processively in the sandwich dimer, resulting in movement apart of NBDs, product release and return to resting state (1). Constant Contact Model. Schematic representation of the Constant Contact Model for the catalytic cycle of ABC transporters. Separate NBD monomers are situated top and bottom in each frame, with the larger core subdomains coloured green and the smaller helical subdomains yellow. (1) ATP is occluded in left site. Opposite site is empty and open by way of outward rotation of its core subdomain, and has low affinity for nucleotide. (2) ATP hydrolysis induces switch to high affinity for ATP in opposite open core subdomain. (3) ATP binds to empty high affinity site. (4) ATP binding to the empty open core subdomain induces its inward rotation and occlusion of ATP. Occlusion of ATP induces opening of opposite site and release of hydrolysis products. (5–6) are repeated steps in the opposite phase with respect to the two active sites to complete the cycle.

**Figure 2:**
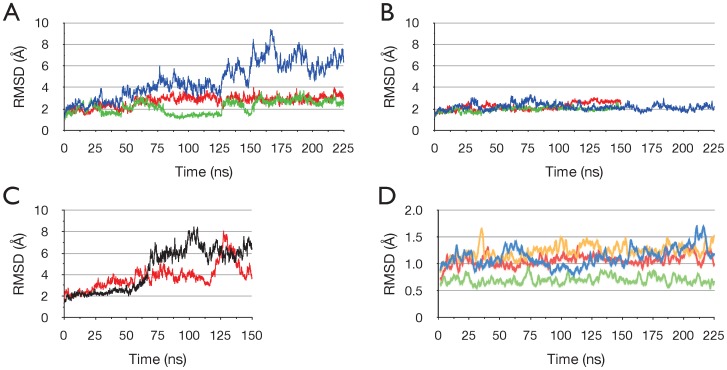
Stability and conformational changes in the simulations. Time series of the RMS deviation of Cα atoms from the starting structure in the ADP+Pi/apo and ATP/apo simulations. Sampled at 75 ps intervals. (A) Simulation ADPPi-1. All Cα atoms blue, monomer A green, monomer B red. (B) ATP/apo simulations. All Cα atoms. ATP-1 blue, ATP-2 green, ATP-3 red. (C) Simulations ADPPi-2 (black) and ADPPi-3 (red). All Cα atoms. (D) Individual core and helical subdomains in simulation ADPPi-1. RMSD moving average with a period of 20. Core A blue, core B yellow, helical A green, helical B red.

The NBDs interact to form two composite active sites, in which the C-motif of one monomer engages nucleotide bound to the Walker A and B sequences of the opposite monomer [20]–[22]. Symmetrical sandwich dimers, in which two nucleotides are bound at the NBD intermonomer interface, have been observed in numerous crystal structures, both of isolated NBDs and whole transporters [Figure 1A; 23–28]. The prevalent view is that the NBD sandwich dimer represents a physiological state in the ABC catalytic cycle, in which the NBDs are proposed to undergo cycles of ATP binding and dimer formation, followed by sequential hydrolysis of both ATPs, and then dimer dissociation, a scheme known as the Processive Clamp or Switch Model [Figure 1B; 13,27,29]. Nevertheless, alternative schemes have been proposed in which the NBDs remain in contact in an asymmetric state, with only one nucleotide tightly bound at any time, referred to as Constant Contact Models [Figure 1C; 2,12, 30–36].

A current question thus involves the nature of the catalytic cycle and the role of the relative movements between the core and helical subdomains. A number of studies of ABC exporters have used agents such as vanadate, beryllium fluoride and aluminium fluoride, which combine with ADP in the active site to form a static analogue of the transition state of the hydrolysis reaction. This arrests the transporter in a state in which one active site is occluded while the other is accessible [37]. MD simulations of the ATP/apo state using the structure of the bacterial ABC exporter Sav1866, which is a homologue of well characterised mammalian MDR ABCs, revealed how an asymmetric closed/open state occurred by way of intrasubunit rotations between the helical and core subdomains [18], [38]. In respect of this, we have proposed a Constant Contact Model in which a closed/open asymmetry between the active sites is mediated by relative rotations between the core and helical subdomains [30], [36].

Recently, a crystal structure of the MJ0796 (E171Q) ABC isolated NBD dimer was published [39]. This dimer was very close in structure to that of the previously reported MJ0796 ATP sandwich dimer [22], but contained the hydrolysis products ADP and phosphate (Pi) in each active site; and is the only published structure of this state for an ABC transporter NBD dimer (Figure 1A). In our Constant Contact Model, the conformation of the ADP+Pi/apo state of the NBD dimer is predicted to be asymmetric, with one site occluded and containing ADP+Pi, and the other open by way of rotation of the empty (apo) core subdomain, primed to bind ATP. In order to investigate the ADP+Pi/apo state, here we performed MD simulations of the isolated NBD dimer using the recent ADP+Pi-bound MJ0796 dimer structure.

## Methods

### System Setup

The starting coordinates for MD simulations were taken from the X-ray structure of MJ0796 (E171Q) (PDB 3TIF; [39]). The E171Q mutation was reversed. Unresolved C-terminal residues 233–235 were modelled using Modeller 8v1 [40]. NBD protomers are referred to as A and B, according to the chain identifiers in the Protein Data Bank entry 3TIF. Two systems were prepared: ADP+Pi/apo and ATP/apo, with the nucleotide bound to the Walker A motif in protomer A. The active site sodium ion was replaced by a magnesium ion. Rotamer A for active site residue Q90 was used. In order to generate starting coordinates for the ATP γ-phosphate moiety, as well as for a canonical water molecule which mediates the interaction of the Walker aspartate and the catalytic magnesium ion, the Cα coordinates of the P-loop of ATP-bound MJ0796 (PDB 1L2T; residues 32–49; [22] were root mean square (RMS) fitted to the equivalent atoms in monomer A in the simulation starting structure. All crystallographic water molecules located within 10 Å of the protein, nucleotide, or Pi group were retained and used in the starting structure.

Each complex was solvated in explicit water using TIP3 water molecules. A truncated octahedral periodic cell was used with a minimum of 20 Å between periodic images of the protein, and the system neutralised with 0.2 M NaCl counterions. Histidine 141 was neutral and protonated at the ε nitrogen and active site histidine 204 was ionized. All other ionisable residues were in the default ionization state, with the exception of E171 in protomer A in the ADP+Pi/apo system, which was neutral.

In order to simulate the ADP+Pi-bound active site, the protonation state of the phosphate ion must be set. In solution at pH 7, the ratio of single to double protonated phosphate is about 2∶3; however the pKa of the ion is likely to be altered by the electrostatic environment in the active site. Whilst a doubly protonated Pi would account for the stoichiometry of the hydrolysis reaction, the exact mechanism by which stoichiometry is maintained with respect to the protons derived from the nucleophilic water is unknown. Default parameters for ATP in MD forcefields, and MD studies of ABCs generally, assume that the γ-phosphate of ATP is unprotonated. In this case, upon nucleophilic attack by an activated water molecule, initially a singly protonated phosphate ion would be formed. Moreover, it is generally expected that in ABC ATPases, the conserved glutamic acid residue, immediately C-terminal to the Walker B aspartate, acts as a general base, receiving a proton from the attacking nucleophile [38].

In view of the above, in the ADP+Pi/apo system, the phosphate was modeled as singly protonated, and the proximal conserved glutamic acid residue as neutral, being protonated at the sidechain carboxyl oxygen proximal to the Pi hydroxyl oxygen. The hydroxyl oxygen of Pi was chosen to be that which, in the crystal structure, is within hydrogen bonding distance of the backbone carbonyl oxygen of D-loop residue A175 from the opposite monomer (Figure S1), thus being consistent with their proximity (2.7 Å). This geometry is also consistent with a canonical hydrolysis scenario [22], [38].

### Simulation Parameters

MD simulations were performed using NAMD version 2.9 [41] with the CHARMM27 force field [42], including φ/ψ cross-term map corrections, and the TIP3P model for water [43]. Partial charges for hydrogen phosphate were from [44]. Unprotonated phosphate oxygens were assigned the CHARMM27 ON3 type, and the hydroxyl oxygen ON4; parameters for the ON3–P-ON3 angle were k = 104.0, θ = 108.23. The SHAKE and SETTLE algorithms were used to constrain bonds containing hydrogens to equilibrium length [45]. A cutoff of 11 Å (switching function starting at 9.5 Å) for van der Waals and real space electrostatic interactions was used. The particle-mesh Ewald method was used to compute long-range electrostatic forces with a grid density of approximately 1/Å^3^ [46]. An integration time step of 1.5 fs was used with a multiple timestepping algorithm; interactions involving covalent bonds and short-range nonbonded interactions were computed every time step, while long-range electrostatic forces were computed every two time steps. A Langevin piston was used to control pressure with a target of 1 atm, a decay period of 100 fs and a damping timescale of 50 fs. Langevin dynamics was utilized to maintain a constant temperature of 343 K with a friction coefficient of 5 ps-1 on all non-hydrogen atoms. The natural habitat of *M. Jannaschii* varies between 48–94°C with an optimal growth temperature of 85°C In a previous MD simulation analysis of MJ0796 [17], we used a temperature of 85°C. Here, a lower temperature of 70°C was used, which was considered to strike a better balance between ensuring conditions that would enable significant conformational changes to occur on the simulated timescale, and the optimal temperature of the MD forcefield.

### Equilibration

The solvated starting structure was minimized using conjugate gradient minimization to a 0.5 kcal/mol.Å RMS gradient during which all non hydrogen atoms of the protein and nucleotide were fixed. Water molecules, NaCl ions, and hydrogens were then further minimized during a 50 ps MD run at 343 K, in which all protein and Mg^2+^ATP/ADP+Pi heavy atoms were again fixed. This starting model was then minimized with harmonic positional constraints on the NCαCO backbone. A 100 kcal/mol Å^2^ force constant was used to minimise the system to a 0.5 kcal/mol Å RMS gradient. The constraints were gradually removed by subsequent minimizations to a 0.1 kcal/mol Å RMS gradient, scaling the initial force constants by factors of 0.5, 0.15, 0.05, and 0. The minimized structure was then heated from 43 K to 343 K in steps of 25 K using velocity reassignment during a 30 ps MD run. Three separate minimizations were performed for each system, using different random seeds for the initial velocity assignments.

### Production Runs

The six equilibrated systems described above were used for the production runs without restraints. For each of the two states, 3 simulations were performed, one for 150 M integration steps (225 ns) and two for 100 M steps (150 ns). All simulations remained stable to completion. For analysis, the coordinates were saved every 5 K steps (7.5 ps).

### Analysis

Principal component analysis of the Cα atom trajectories was performed using the GROMACS package, with frames taken at 0.075 ns intervals over the entire trajectory, and aligned to the starting coordinates [47]. The program Hingefinder [48] was used to analyse domain rotations: this analysis used Cα coordinates, the slow partitioning algorithm and a tolerance of 40%. VMD [49], Xplor-NIH [50] and Simulaid [51] were used to prepare the system and analyse MD trajectories. Calculation of the RMSD of individual core subdomains used Cα atom coordinates of residues 1–90 and 164–228; this excludes a highly mobile unstructured C-terminal tail of 7 residues (residues 229–235). Calculation of the RMSD of individual helical subdomains used Cα atom coordinates of residues 94–111 and 117–161; this excludes the α3-4 loop (residues 112–116) joining the first and second α-helices in the helical subdomain (see Results). These calculations also exclude the Q-loop (91–93) and Pro-loop (161–163), which are not assigned to either subdomain. All structural figures were prepared using PyMol (http://www.pymol.org/pymol), except for Figure S2, which was prepared using VMD.

## Results

To investigate the catalytic cycle of the ABC transporter NBDs, MD simulations of the ADP+Pi/apo state of the MJ0796 dimer were performed, using the recent ADP+Pi-bound MJ0796 dimer structure. Simulations of the ATP/apo state were also performed, under the same conditions. Three unrestrained simulations of each state were performed, one for 150 M integration steps (225 ns) and two for 100 M steps (150 ns), totaling six independent simulations, each originating from independently equilibrated starting structures. The simulations of the ADP+Pi/apo state are referred to as ADPPi-1, ADPPi-2 and ADPPi-3 for the 225 ns and two 150 ns runs respectively, and the simulations of the ATP/apo state are referred to as ATP-1, ATP-2 and ATP-3 for the 225 ns and two 150 ns runs, respectively.

To assess the stability of the protein, Figure 2 shows the time series of the RMS deviation (RMSD) of Cα atom coordinates from the initial structure, for all simulations. In Figure 2A the RMSD for run ADPPi-1 is plotted; this indicates that the dimer undergoes a relatively large change with respect to the crystal structure. Also plotted in Figure 2A is the RMSD for each NBD monomer separately; this shows that the monomers remain relatively close to the starting structure. Thus, together the data in Figure 2A indicate that the large RMSD observed in the dimer is due to relative movements between the monomers. Figure 2B shows that the large conformational change that occurred in run ADPPi-1, did not occur in any of the simulations of the ATP/apo state, which remained stable and relatively close to the starting structure. Figure 2C shows, in contrast, that in the two separate 150 ns runs of the ADP+Pi/apo state, relatively large changes in RMSD occurred, as in run ADPPi-1.

In order to investigate the stability of the protein more closely, Figure 2D shows the time series of the RMSD for the individual core and helical subdomains in each NBD for run ADPPi-1. The calculation for the helical subdomains excludes the five residue loop joining the first and second α-helices in the helical subdomain (α3-4 loop, residues 112–116), which varies in structure between the monomers in the crystal structure, and was observed to be highly mobile in MD simulations [17], [52]. This analysis shows that these subdomains remain very close to the starting conformation; these data, and in particular the very low RMSD for the monomer A helical subdomain, indicate clearly that the high temperature of the simulation (70°C) did not induce significant instability in the protein.

**Figure 3:**
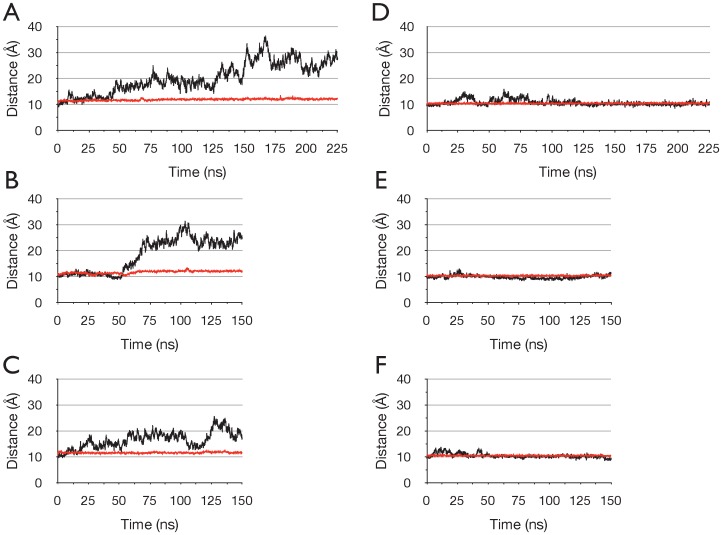
Opening of the empty active site in the ADP+Pi/apo dimer. Changes in the active sites monitored by the distance between the geometric centres of the Cα atoms of Walker A residues 43–46 (GKST) and C-motif residues 147–150 (SGGQ) of opposing monomers. (A-C) show ADPPi runs 1–3 respectively, (D-F) show ATP runs 1–3 respectively. Nucleotide-bound active site is red and empty active site black. Sampled at 75 ps intervals.

Visual examination of the simulation trajectories revealed that in the three simulations of the ADP+Pi/apo state, the empty active site opened, while the ADP+Pi-bound site remained closed. Opening of either active site did not occur in any of the three ATP/apo runs. This is illustrated in Figure 3, where the time series of the distances between opposing helical and core subdomains are plotted for each simulation.

To further define the conformational changes that occurred in the simulations of the ADP+Pi/apo state, Principal Component Analysis (PCA) of the Cα atom coordinate trajectories was performed. PCA distinguishes concerted global motions from random thermal fluctuations, through analysis of pairwise correlations between atoms, over a simulation trajectory. The derived Principal Components (PCs) are ranked according to the amplitude of the motions they describe.

**Figure 4:**
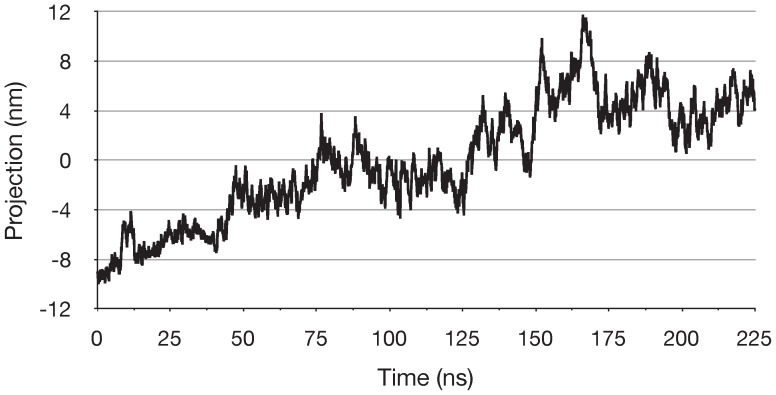
Global change in the ADP+Pi/apo simulation. Projection of the Cα atom trajectory for run ADPPi-1 onto PCA mode 1. Sampled at 75 ps intervals.

For run ADPPi-1, the first PCA mode (PC1) represented 60% of all Cα atom fluctuations, while PC2 and PC3 represented 11.4% and 5.5% respectively; PC1 thus describes a clearly dominant motion. Figure 4 shows the time series of the projection of the Cα atom trajectory on to PC1 derived from run ADPPi-1. The projection illustrates the extent to which the protein has moved along the transition described by the mode; Figure 4 thus depicts a transition of the protein from one conformation toward another. For run ADPPi-1, the time series of the projection of PC1 correlated with the time series of the distance between the opposing helical and core subdomains comprising the empty active site (Figure 3), with a correlation coefficient of 0.991; this indicates that PC1 embodies the global motion by which the empty active site opens.

**Figure 5:**
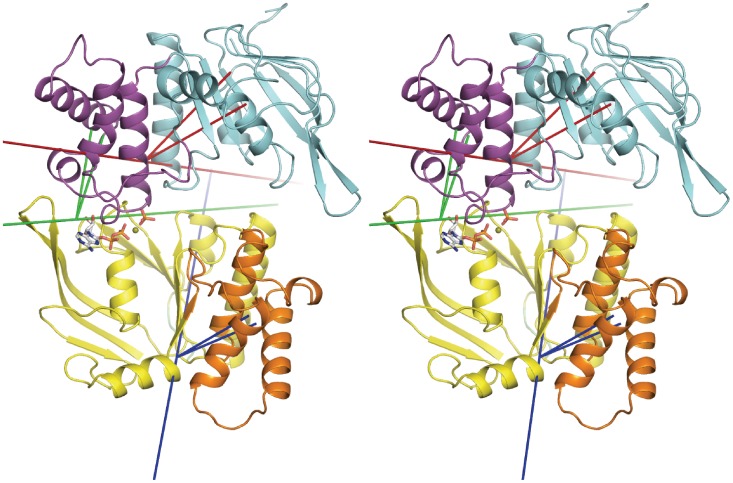
Opening of the empty active site via relative movements of core and helical subdomains. Stereo view of the trajectory frame corresponding to the maximum projection of PC1 in run ADPPi-1, showing rotating domains and their hinge axes as defined by the Hingefinder analysis of the maximum and minimum projection structures of PC1. Axes are longer red, blue and green lines, with the two shorter same-coloured lines connecting the centre of mass of the rotating domain to the pivot point on the axis. Cartoon image of the protein with monomer A bottom and monomer B top. Rotating core subdomain of monomer B (residues 1–88, 165–174, 180–234) is coloured cyan and its hinge axis is in red. Rotating helical subdomain of monomer B (residues 90–164) is coloured magenta and its hinge axis is in green. Rotating helical subdomain of monomer A (residues 87–163) is coloured orange and its hinge axis is in blue. Rotating domain residues are as determined by Hingefinder.

To characterise PC1 from run ADPPi-1 in terms of domain motions, the program Hingefinder was used to identify rotating domains between the maximum and minimum projection structures of PC1; these structures represent the two extremes between which the transition described by the mode occurs. Figure 5 shows the trajectory frame corresponding to the maximum projection structure for PC1, which occurs at t = 167 ns. This illustrates the open apo site, as well the rotating domains in PC1, as detected and defined by the analysis, and their rotation axes and pivot points, as well as the distance moved by the geometric centre of the respective rotating domain.

This analysis reveals that the largest change is undergone by the core subdomain of monomer B, which rotates outward and away from the opposite helical subdomain, thereby opening the unliganded active site (Figure 5; Figure S2). In addition, a rotation of the monomer A helical subdomain contributes to opening of the empty active site. The helical subdomain that engages the nucleotide also pivots, changing its orientation with respect to the opposite P-loop. Nevertheless, interactions of the nucleotide with the conserved serine and glutamine residues of the monomer B LSGGQ C-motif, present in the crystal structure, are essentially maintained in the simulation (Figure S3).

**Figure 6:**
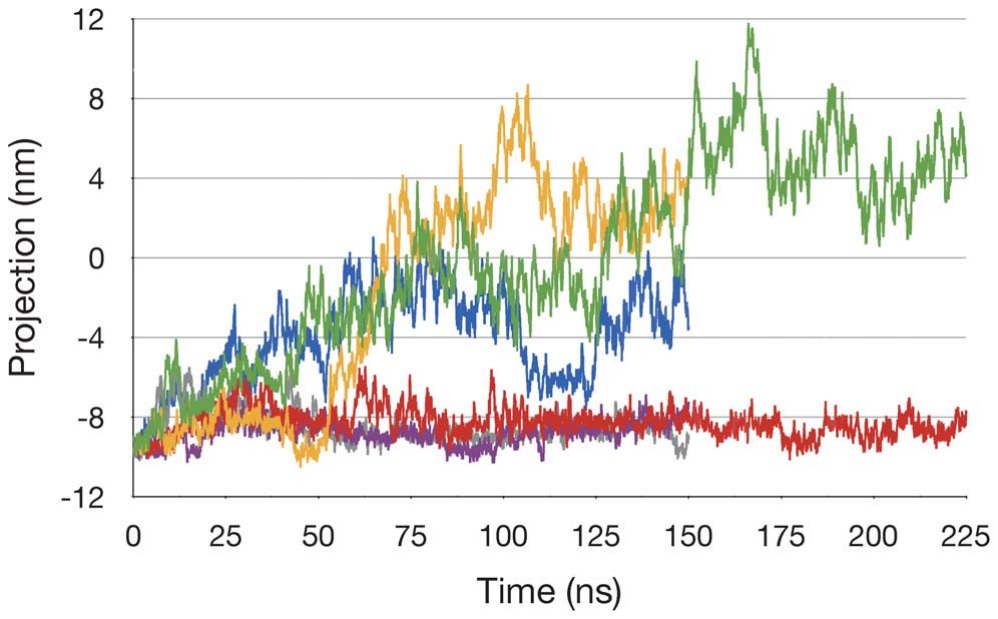
Extent to which the predominant global changes in run ADP+Pi-1 occurs in the other simulations. Projection of PCA mode 1 derived from run ADP+Pi-1 onto the Cα atom trajectory for each run. ADPPi-1 green, ADPPi-2 yellow, ADPPi-3 blue, ATP-1 red, ATP-2 mauve, ATP-3 grey. The sign for the values for run ADPPi-1 are reversed with respect to Figure 4, to facilitate the comparison. Sampled at 75 ps intervals.

To examine the other simulations with respect to the dominant motion observed in run ADPPi-1, the Cα atom coordinate trajectory from each simulation was projected onto PC1 from run ADPPi-1 (Figure 6). This shows that, while the two 150 ns runs of the ADP+Pi/apo dimer sampled the configurational space defined by this mode to a significant extent, comparatively, the ATP/apo runs clearly did not. The degree to which the other simulations sampled the configurational space defined by PC1 from run ADPPi-1 was also examined by calculating the subspace overlap coefficients between this mode and PCs 1–2 calculated from the Cα atom coordinate trajectory of each simulation: these were- ADPPi-2, 0.819; ADPPi-3, 0.804; ATP-1, 0.296; ATP-2, 0.011; ATP-3, 0.381. This further indicates that the configurational space sampled in runs ADPPi-2 and ADPPi-3 clearly overlapped with that of run ADPPi-1, while simulations of the ATP-bound dimer did so to a significantly lesser degree.

Notably, for run ADPPi-3, PCs 1 and 2 corresponded approximately to PCs 2 and 1, respectively, from run ADPPi-1. This indicates that for run ADPPi-3, the mode described by ADPPi-1 PC1 was not the largest amplitude motion that occurred. This, however, is consistent with the observation that opening of the active site in ADPPi-3 did not occur to as great an extent as in the other two ADPPi runs. Finally, the correlation coefficients between the distance the active site opened, and the projection onto ADPPi-1 PC1 were 0.995 for run ADPPi-2 and 0.818 for run ADPPi-3, further illustrating that motions described by PC1 from run ADPPi-1 were well correlated with active site opening in runs ADPPi-2 and ADPPi-3.

## Discussion

In this study, to further understand and characterise the functioning of the ABC ATPase, unrestrained MD simulations of the ADP+Pi/apo and ATP/apo states of the isolated NBD dimer of the bacterial ABC transporter MJ0796 were performed. In three independent simulations of the ADP+Pi/apo state, comprising a total of >0.5 μs of simulated time, the empty active site opened, clearly sufficient to allow binding of nucleotide to the unliganded Walker A motif. This opening was achieved substantially by way of rotation of the empty core subdomain away from the opposite monomer. Rotation of the helical subdomain engaging the empty active site also contributed toward its opening. ADP+Pi remained bound in the active site, and the LSGGQ motif maintained contact with the nucleotide, throughout all simulations of the ADP+Pi/apo state. In contrast, in three independent simulations of the ATP/apo state, also comprising a total of >0.5 μs of simulated time, the core and helical subdomains forming the empty active site did not alter in conformation from the sandwich dimer starting structure, and no opening of either active site was observed.

The fact that the empty active site did not open in the simulations of the ATP/apo state suggests that active site opening in the ADP+Pi/apo state was not a random fluctuation. This is also supported by the reproducibility of the opening as shown by the PCA data (Figure 6). Crystallographic and MD simulation analyses of the ABC NBD monomer suggested that conformational changes involving the helical and core subdomains, between the ATP-bound, and ADP-bound or empty NBDs, are determined simply by the nature of the nucleotide, or its absence, in the Walker A and B motifs [15], [17], [53]. However, here we find that removal of nucleotide from one monomer did not result in conformational changes in the ATP/apo state. This indicates that the outward rotation of the core subdomain observed in the ADP+Pi/apo state was contingent upon hydrolysis products being in the active site in which the unliganded monomer C-motif engages. Thus the apo NBD monomer also utilized the C-motif to sense and respond dynamically to events in the opposite core subdomain.

The opening of the empty active site in the ADP+Pi/apo state is consistent with our Constant Contact Model, with the conformation observed here corresponding well to stages 2 and 5 in Figure 1C. It is also consistent with experimental data showing that in the vanadate trapped state, which is expected to be a transition state analogue close to the ADP+Pi state, one site is occluded while the other is accessible [37]. However, the fact that the empty active site did not open in the ATP/apo state does not appear consistent with our model, which predicts the empty site to be open (stages 1 and 4 in Figure 1C).

In regard to the ATP/apo result, it should be noted that the Constant Contact Model is based simply on two premises [30]: an alternating function between the active sites, and a sequence for each active site as follows- empty (open, high affinity), ATP-bound (open), ATP-bound (closed), ADP+Pi (closed), ADP+Pi (open), empty (open, low affinity). The important feature of the ATP/empty state, predicted on this basis, is that the empty active site has low affinity. Thus, it may be that the active site opens to release ADP+Pi, and then closes to produce a low affinity site. However, in our MD simulations of the ATP/apo state of the Sav1866 cytosolic domains, the empty active site opened, notably, however, to a lesser extent than observed here for the ADP+Pi simulations [18], [38].

How might the data from our MD studies be reconciled with the Constant Contact Model? Studies of ABCB1, nucleotide-trapped using different transition state analogues, suggested that the degree of opening of the opposite, non-occluded active site is a function of the point along the reaction coordinate that is mimicked by the particular transition state analogue [37]. Moreover, it can be inferred that the ABC transporter undergoes a significant global transition that pivots about the hydrolysis reaction transition state [2]. In our simulations of the Sav1866 ATP/apo state, a progression toward a putative hydrolysis-competent state was observed, whereas in the present simulations, this did not occur. Thus, our MD data are consistent with the notion that in the ATP/apo state, the opening of the empty active is dependent on the position of the ATP-bound site along the hydrolysis reaction continuum. Furthermore, since the Sav1866 system included the TMD intracytosolic domains, together our MD data are consistent with the idea that the TMDs propagate signals which stimulate hydrolysis, and that in their absence, the empty active site remains closed, corresponding to the ground or reactant state of the opposite ATP-bound site. This is supported by the generally low ATP hydrolysis rates exhibited by isolated ABC NBDs compared to full transporters.

On the basis of the present and previous data, we thus propose a modification to our model such that, following ADP+Pi release, the empty active site is closed in the ATP/apo state, producing a low affinity apo site. From this ATP/apo state, the empty site opens along a continuum that is a function of the point the ATP-bound site is along the hydrolysis reaction coordinate, becoming fully open in the ADP+Pi/apo state.

It might also be argued that the present results support the Switch Model. Thus, the stability of the ATP/apo simulation system, and the observed opening in the ADP+Pi/apo system, are consistent with the central notions of the Switch Model that ATP stabilises the active site while the post-hydrolytic state destabilizes it. It is important to note, however, that the ATP/apo and ADP+Pi/apo states per se are not part of the Switch Model, so the interpretation of the results in terms of the Switch Model is not clear. Nevertheless, some relevant questions do arise. Thus, how might the NBD dimer’s apparent ability to exchange nucleotide at one active site, before the NBD protomers dissociate, be reconciled with the Switch Model?

The salient feature of the results of this study is the ability of the NBD dimer to respond in a dramatically different way to the presence of either ATP, or the hydrolysis products ADP+Pi, within the active site. This shows that the protein is able to detect and respond to relatively small changes in the nucleotide occupancy of the active site. This is in accord with the notion that ATP hydrolysis induces conformational changes that, in turn, are transmitted to the TMDs, resulting in substrate translocation.

The present findings also suggest that if the conformational differences between the two states observed here indeed embody functional changes, then the free energy released upon ATP hydrolysis may not be transmitted directly to the protein to produce a high energy state, that then relaxes via a functional transition to perform the mechanical work of substrate translocation. Rather, it suggests that the equilibrated, or lowest energy conformation of the protein is different for any particular state of the transport cycle, with conformational changes between states corresponding to those that implement the translocation process. In this view, conformational changes between states occur through thermal fluctuations and are reversible; and the vectorial nature of the transport cycle is determined simply by the energy gradient for ATP hydrolysis.

## Supporting Information

Figure S1 **Structure of the ADP+Pi-bound active site in the MJ0796 NBD Dimer.** Structural figure prepared from PDB entry 3TIF, used as the starting structure in the simulations. Orientation of the monomers is approximately as in Figure 1A; as are the stick forms and colours of the nucleotide and sidechains. Backbone atoms of the C-motif (left) and D-loop (right) of the trans monomer are shown in stick form, with carbon green, oxygen red, and nitrogen blue. A hydrogen bond between the backbone carbonyl oxygen of D-loop residue A175 and the Pi molecule is shown as a red dashed line. Weaker interactions between the conserved glutamine (left) and the E171Q glutamine (centre) are shown as green dashed lines.
(PDF)

Figure S2 **PCA mode 1 from simulation ADPPi-1.** (A) to illustrate the mode, the maximum and minimum projections of PC1 along the trajectory, together with 8 interpolated structures, are superimposed by rms fitting using all Cα atom coordinates. Cα atom coordinates only are shown, with core subdomains blue and helical subdomains green. Red arrows indicate P-loops and black arrows the LSGGQ motif. (B) and (C) stereo views derived from (A) using the trajectory smoothing function in VMD to reduce range of motion and illustrate relative degrees of motion. Arrows as in (A). Colours indicate progression in time with blue near t = 0 and red near t = 150 ns.
(TIFF)

Figure S3 **Interaction of the C-motif with the nucleotide in the ADP+Pi/apo simulations.** (A-C) Time series of the distance between the C-motif serine hydroxyl oxygen and the proximal nucleotide β-phosphate oxygen for ADPPi runs 1–3 respectively. (D-F) Time series of the minimum distance between either the oxygen or nitrogen of the C-motif glutamine (LSGGQ) sidechain amido, and the proximal nucleotide ribose hydroxyl oxygen, for ADPPi runs 1–3 respectively.
(PDF)

## References

[1] Holland IB, Blight MA (1999) ABC-ATPases, adaptable energy generators fuelling transmembrane movement of a variety of molecules in organisms from bacteria to humans. J Mol Biol 293: 381–399. 10529352 10.1006/jmbi.1999.2993

[2] Jones PM, George AM (2004) The ABC transporter structure and mechanism: perspectives on recent research. Cell Mol Life Sci 61: 1–18. 15052411 10.1007/s00018-003-3336-9 PMC11138499

[3] Dean M, Rzhetsky A, Allikmets R (2001) The human ATP-binding cassette (ABC) transporter superfamily. Genome Res 11: 1156–1166. 11435397 10.1101/gr.184901

[4] Eckford PD, Sharom FJ (2009) ABC efflux pump-based resistance to chemotherapy drugs. Chem Rev 109: 2989–3011. 19583429 10.1021/cr9000226

[5] Ernst R, Kueppers P, Stindt J, Kuchler K, Schmitt L (2010) Multidrug efflux pumps: substrate selection in ATP-binding cassette multidrug efflux pumps–first come, first served? FEBS J 277: 540–549. 19961541 10.1111/j.1742-4658.2009.07485.x

[6] Kerr ID, Jones PM, George AM (2010) Multidrug efflux pumps: The structures of prokaryotic ATP-binding cassette transporter efflux pumps and implications for our understanding of eukaryotic P-glycoproteins and homologues. FEBS Journal 277: 550–563. 19961540 10.1111/j.1742-4658.2009.07486.x

[7] Riordan JR, Rommens JM, Kerem B, Alon N, Rozmahel R, et al (1989) Identification of the cystic fibrosis gene: cloning and characterization of complementary DNA. Science 245: 1066–1073. 2475911 10.1126/science.2475911

[8] Gadsby DC, Vergani P, Csanady L (2006) The ABC protein turned chloride channel whose failure causes cystic fibrosis. Nature 440: 477–483. 16554808 10.1038/nature04712 PMC2720541

[9] Davidson AL, Dassa E, Orelle C, Chen J (2008) Structure, function, and evolution of bacterial ATP-binding cassette systems. Microbiol Mol Biol Rev 72: 317–364, table of contents. 10.1128/MMBR.00031-07 PMC2415747 18535149

[10] Higgins CF (1992) ABC transporters; from microorganisms to man. Annu Rev Cell Biol 8: 67–113. 1282354 10.1146/annurev.cb.08.110192.000435

[11] Hyde SC, Emsley P, Hartshorn MJ, Mimmack MM, Gileadi U, et al (1990) Structural model of ATP-binding proteins associated with cystic fibrosis, multidrug resistance and bacterial transport. Nature 346: 362–365. 1973824 10.1038/346362a0

[12] Jones PM, George AM (2002) Mechanism of ABC transporters: a molecular dynamics simulation of a well characterized nucleotide-binding subunit. Proc Natl Acad Sci U S A 99: 12639–12644. 12237398 10.1073/pnas.152439599 PMC130513

[13] Higgins CF, Linton KJ (2004) The ATP switch model for ABC transporters. Nat Struct Mol Biol 11: 918–926. 15452563 10.1038/nsmb836

[14] George AM, Jones PM (2012) Perspectives on the structure-function of ABC transporters: the Switch and Constant Contact models. Prog Biophys Mol Biol 109: 95–107. 22765920 10.1016/j.pbiomolbio.2012.06.003

[15] Yuan YR, Blecker S, Martsinkevich O, Millen L, Thomas PJ, et al (2001) The crystal structure of the MJ0796 ATP-binding cassette. Implications for the structural consequences of ATP hydrolysis in the active site of an ABC transporter. J Biol Chem 276: 32313–32321. 11402022 10.1074/jbc.M100758200

[16] Campbell JD, Deol SS, Ashcroft FM, Kerr ID, Sansom MS (2004) Nucleotide-dependent conformational changes in HisP: molecular dynamics simulations of an ABC transporter nucleotide-binding domain. Biophys J 87: 3703–3715. 15377525 10.1529/biophysj.104.046870 PMC1304884

[17] Jones PM, George AM (2007) Nucleotide-dependent allostery within the ABC transporter ATP-binding cassette: a computational study of the MJ0796 dimer. J Biol Chem 282: 22793–22803. 17485460 10.1074/jbc.M700809200

[18] Jones PM, George AM (2011) Molecular-dynamics simulations of the ATP/apo state of a multidrug ATP-binding cassette transporter provide a structural and mechanistic basis for the asymmetric occluded state. Biophys J 100: 3025–3034. 21689537 10.1016/j.bpj.2011.05.028 PMC3123983

[19] Orelle C, Alvarez FJ, Oldham ML, Orelle A, Wiley TE, et al (2010) Dynamics of alpha-helical subdomain rotation in the intact maltose ATP-binding cassette transporter. Proc Natl Acad Sci U S A 107: 20293–20298. 21059948 10.1073/pnas.1006544107 PMC2996640

[20] Jones PM, George AM (1999) Subunit interactions in ABC transporters: towards a functional architecture. FEMS Microbiol Lett 179: 187–202. 10518715 10.1111/j.1574-6968.1999.tb08727.x

[21] Hopfner KP, Karcher A, Shin DS, Craig L, Arthur LM, et al (2000) Structural biology of Rad50 ATPase: ATP-driven conformational control in DNA double-strand break repair and the ABC-ATPase superfamily. Cell 101: 789–800. 10892749 10.1016/s0092-8674(00)80890-9

[22] Smith PC, Karpowich N, Millen L, Moody JE, Rosen J, et al (2002) ATP binding to the motor domain from an ABC transporter drives formation of a nucleotide sandwich dimer. Mol Cell 10: 139–149. 12150914 10.1016/s1097-2765(02)00576-2 PMC3516284

[23] Zaitseva J, Oswald C, Jumpertz T, Jenewein S, Wiedenmann A, et al (2006) A structural analysis of asymmetry required for catalytic activity of an ABC-ATPase domain dimer. Embo J 25: 3432–3443. 16858415 10.1038/sj.emboj.7601208 PMC1523178

[24] Procko E, Ferrin-O’Connell I, Ng SL, Gaudet R (2006) Distinct structural and functional properties of the ATPase sites in an asymmetric ABC transporter. Mol Cell 24: 51–62. 17018292 10.1016/j.molcel.2006.07.034

[25] Dawson RJ, Locher KP (2006) Structure of a bacterial multidrug ABC transporter. Nature 443: 180–185. 16943773 10.1038/nature05155

[26] Ward A, Reyes CL, Yu J, Roth CB, Chang G (2007) Flexibility in the ABC transporter MsbA: Alternating access with a twist. Proc Natl Acad Sci U S A 104: 19005–19010. 18024585 10.1073/pnas.0709388104 PMC2141898

[27] Chen J, Lu G, Lin J, Davidson AL, Quiocho FA (2003) A tweezers-like motion of the ATP-binding cassette dimer in an ABC transport cycle. Mol Cell 12: 651–661. 14527411 10.1016/j.molcel.2003.08.004

[28] Oldham ML, Chen J (2011) Crystal structure of the maltose transporter in a pretranslocation intermediate state. Science 332: 1202–1205. 21566157 10.1126/science.1200767

[29] Janas E, Hofacker M, Chen M, Gompf S, van der Does C, et al (2003) The ATP hydrolysis cycle of the nucleotide-binding domain of the mitochondrial ATP-binding cassette transporter Mdl1p. J Biol Chem 278: 26862–26869. 12746444 10.1074/jbc.M301227200

[30] Jones PM, George AM (2009) Opening of the ADP-bound active site in the ABC transporter ATPase dimer: evidence for a constant contact, alternating sites model for the catalytic cycle. Proteins 75: 387–396. 18831048 10.1002/prot.22250

[31] Jones PM, O’Mara ML, George AM (2009) ABC transporters: a riddle wrapped in a mystery inside an enigma. Trends Biochem Sci 34: 520–531. 19748784 10.1016/j.tibs.2009.06.004

[32] Sauna ZE, Kim IW, Nandigama K, Kopp S, Chiba P, et al (2007) Catalytic cycle of ATP hydrolysis by P-glycoprotein: evidence for formation of the E.S reaction intermediate with ATP-gamma-S, a nonhydrolyzable analogue of ATP. Biochemistry 46: 13787–13799. 17988154 10.1021/bi701385t

[33] Loo TW, Bartlett MC, Clarke DM (2010) Human P-glycoprotein is active when the two halves are clamped together in the closed conformation. Biochem Biophys Res Commun 395: 436–440. 20394729 10.1016/j.bbrc.2010.04.057

[34] Siarheyeva A, Liu R, Sharom FJ (2010) Characterization of an asymmetric occluded state of P-glycoprotein with two bound nucleotides: implications for catalysis. J Biol Chem 285: 7575–7586. 20061384 10.1074/jbc.M109.047290 PMC2844205

[35] Verhalen B, Wilkens S (2011) P-glycoprotein retains drug-stimulated ATPase activity upon covalent linkage of the two nucleotide binding domains at their C-terminal ends. J Biol Chem 286: 10476–10482. 21278250 10.1074/jbc.M110.193151 PMC3060501

[36] Jones PM, George AM (2013) Mechanism of the ABC transporter ATPase domains: catalytic models and the biochemical and biophysical record. Crit Rev Biochem Mol Biol 48: 39–50. 23131203 10.3109/10409238.2012.735644

[37] Russell PL, Sharom FJ (2006) Conformational and functional characterization of trapped complexes of the P-glycoprotein multidrug transporter. Biochem J 399: 315–323. 16803457 10.1042/BJ20060015 PMC1609918

[38] Jones PM, George AM (2012) Role of the D-loops in allosteric control of ATP hydrolysis in an ABC transporter. J Phys Chem A 116: 3004–3013. 22369471 10.1021/jp211139s

[39] Zoghbi ME, Fuson KL, Sutton RB, Altenberg GA (2012) Kinetics of the association/dissociation cycle of an ATP-binding cassette nucleotide-binding domain. J Biol Chem 287: 4157–4164. 22158619 10.1074/jbc.M111.318378 PMC3281709

[40] Sali A, Blundell TL (1993) Comparative protein modelling by satisfaction of spatial restraints. J Mol Biol 234: 779–815. 8254673 10.1006/jmbi.1993.1626

[41] Kale L, Skeel R, Bhandarkar M, Brunner R, Gursoy A, et al (1999) NAMD2: Greater Scalability for Parallel Molecular Dynamics. Journal of Computational Physics 151: 283–312.

[42] MacKerell AD, Bashford D, Bellott M, Dunbrack RL, Evanseck JD, et al (1998) All-Atom Empirical Potential for Molecular Modeling and Dynamics Studies of Proteins. The Journal of Physical Chemistry B 102: 3586–3616. 24889800 10.1021/jp973084f

[43] Jorgensen WL, Chandrasekhar J, Madura JD, Impey RW, Klein ML (1983) Comparison of simple potential functions for simulating liquid water. The Journal of Chemical Physics 79: 926–935.

[44] Wriggers W, Schulten K (1997) Stability and dynamics of G-actin: back-door water diffusion and behavior of a subdomain 3/4 loop. Biophys J 73: 624–639. 9251782 10.1016/S0006-3495(97)78098-6 PMC1180962

[45] Ryckaert J-P, Ciccotti G, Berendsen HJC (1977) Numerical integration of the cartesian equations of motion of a system with constraints: molecular dynamics of n-alkanes. Journal of Computational Physics 23: 327–341.

[46] Darden T, York D, Pedersen L (1993) Particle mesh Ewald: An N [center-dot] log(N) method for Ewald sums in large systems. The Journal of Chemical Physics 98: 10089–10092.

[47] Lindahl E, Hess B, van der Spoel D (2001) GROMACS 3.0: a package for molecular simulation and trajectory analysis. Journal of Molecular Modeling 7: 306–317.

[48] Wriggers W, Schulten K (1997) Protein domain movements: detection of rigid domains and visualization of hinges in comparisons of atomic coordinates. Proteins 29: 1–14. 9294863

[49] Humphrey W, Dalke A, Schulten K (1996) VMD: visual molecular dynamics. J Mol Graph 14: 33–38, 27–38. 10.1016/0263-7855(96)00018-5 8744570

[50] Schwieters CD, Kuszewski JJ, Tjandra N, Clore GM (2003) The Xplor-NIH NMR molecular structure determination package. J Magn Reson 160: 65–73. 12565051 10.1016/s1090-7807(02)00014-9

[51] Mezei M (1997) Optimal Position of the Solute for Simulations. Journal of Computational Chemistry 18: 812–815.

[52] Oliveira AS, Baptista AM, Soares CM (2010) Insights into the molecular mechanism of an ABC transporter: conformational changes in the NBD dimer of MJ0796. J Phys Chem B 114: 5486–5496. 20369870 10.1021/jp905735y

[53] Karpowich N, Martsinkevich O, Millen L, Yuan YR, Dai PL, et al (2001) Crystal structures of the MJ1267 ATP binding cassette reveal an induced-fit effect at the ATPase active site of an ABC transporter. Structure (Camb) 9: 571–586. 11470432 10.1016/s0969-2126(01)00617-7

