# Supplementary figures and images for: An Asymmetric Post-Hydrolysis State of the ABC Transporter ATPase Dimer
Source: PLoS One. 2013 Apr 3;8(4):e59854. doi: 10.1371/journal.pone.0059854 (PMC3616075; doi:10.1371/journal.pone.0059854)

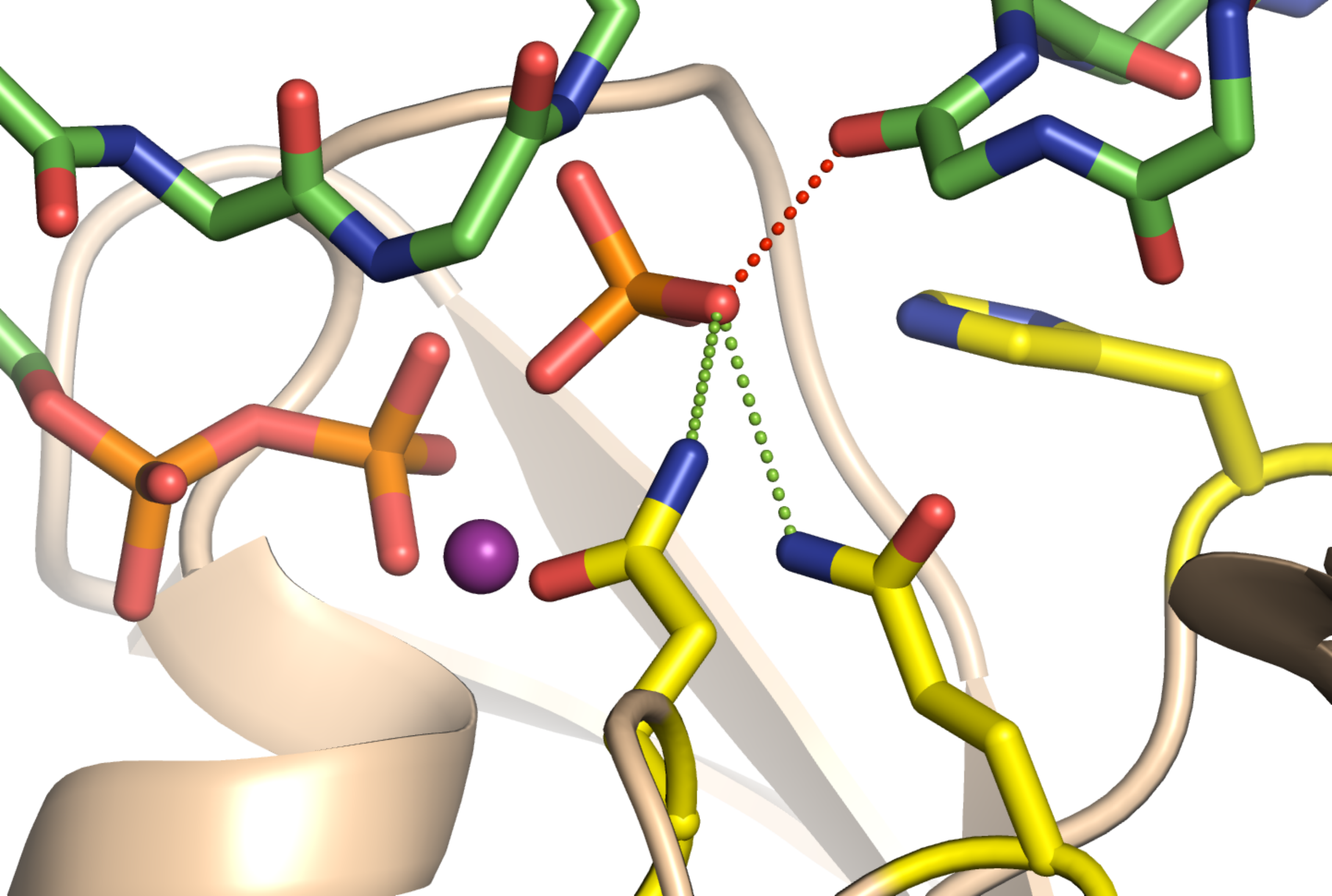

Supplement: Figure S1 — Structure of the ADP+Pi-bound active site in the MJ0796 NBD Dimer. Structural figure prepared from PDB entry 3TIF, used as the starting structure in the simulations. Orientation of the monomers is approximately as in Figure 1A; as are the stick forms and colours of the nucleotide and sidechains. Backbone atoms of the C-motif (left) and D-loop (right) of the trans monomer are shown in stick form, with carbon green, oxygen red, and nitrogen blue. A hydrogen bond between the backbone carbonyl oxygen of D-loop residue A175 and the Pi molecule is shown as a red dashed line. Weaker interactions between the conserved glutamine (left) and the E171Q glutamine (centre) are shown as green dashed lines. (PDF) [file pone.0059854.s001.pdf]

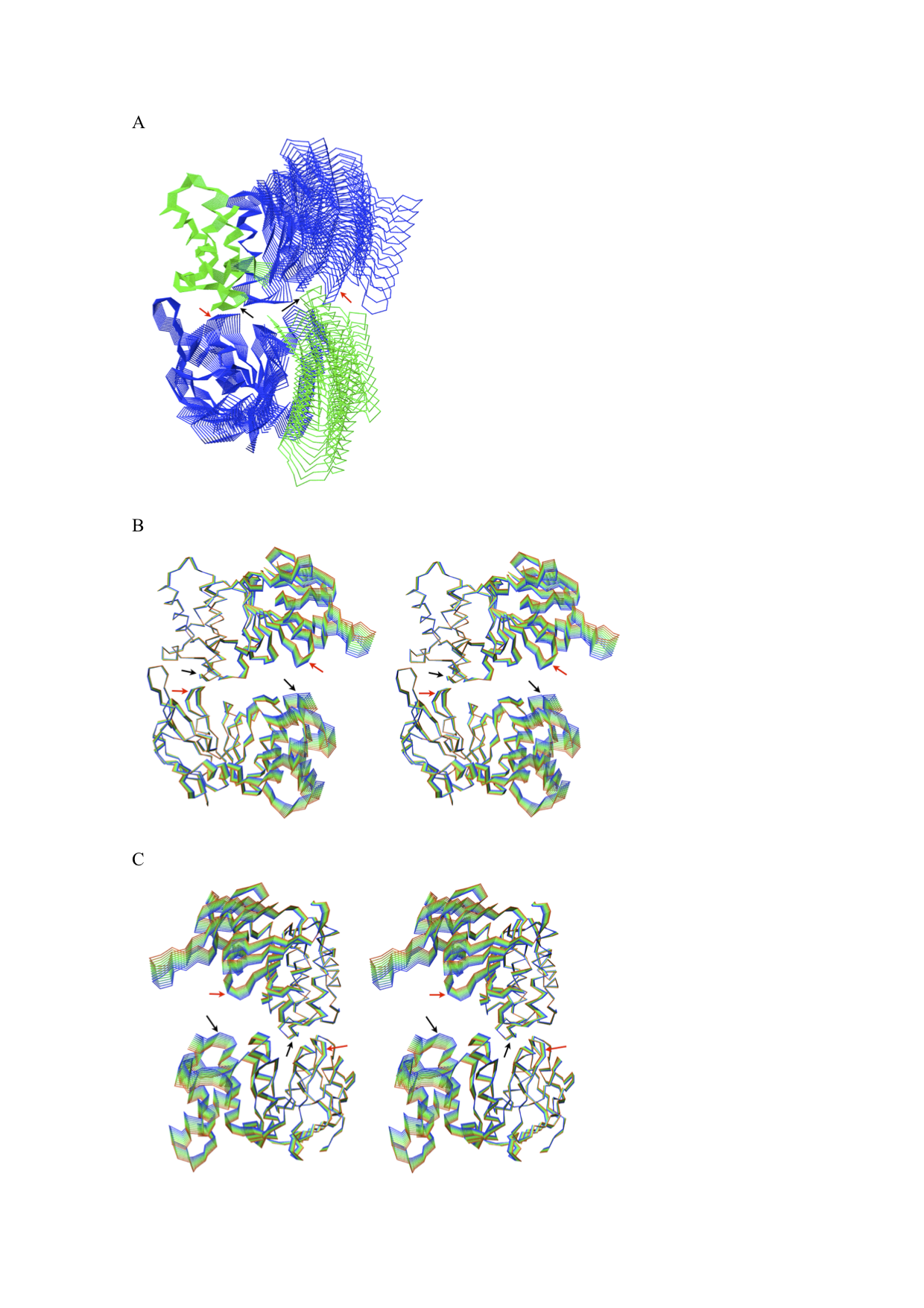

Supplement: Figure S2 — PCA mode 1 from simulation ADPPi-1. (A) to illustrate the mode, the maximum and minimum projections of PC1 along the trajectory, together with 8 interpolated structures, are superimposed by rms fitting using all Cα atom coordinates. Cα atom coordinates only are shown, with core subdomains blue and helical subdomains green. Red arrows indicate P-loops and black arrows the LSGGQ motif. (B) and (C) stereo views derived from (A) using the trajectory smoothing function in VMD to reduce range of motion and illustrate relative degrees of motion. Arrows as in (A). Colours indicate progression in time with blue near t = 0 and red near t = 150 ns. (TIFF) [file pone.0059854.s002.tif]

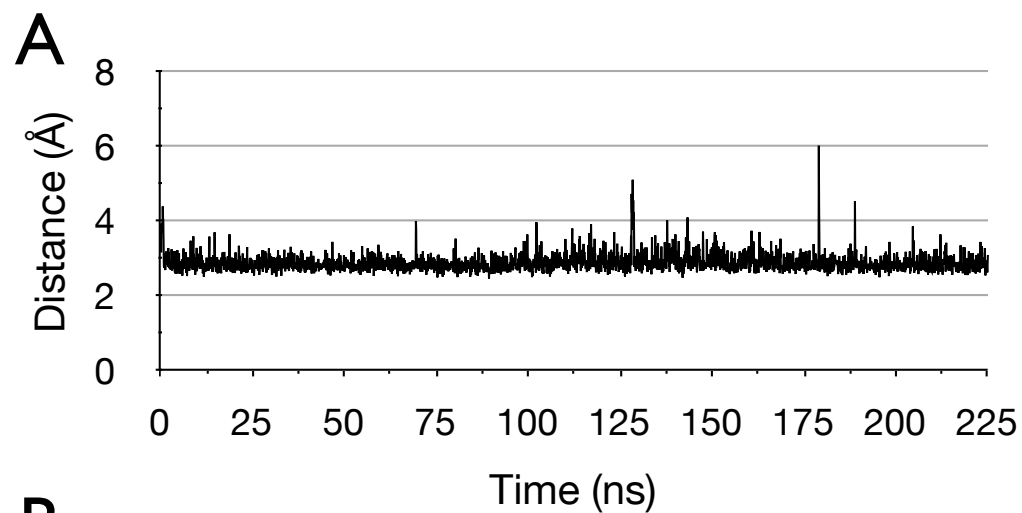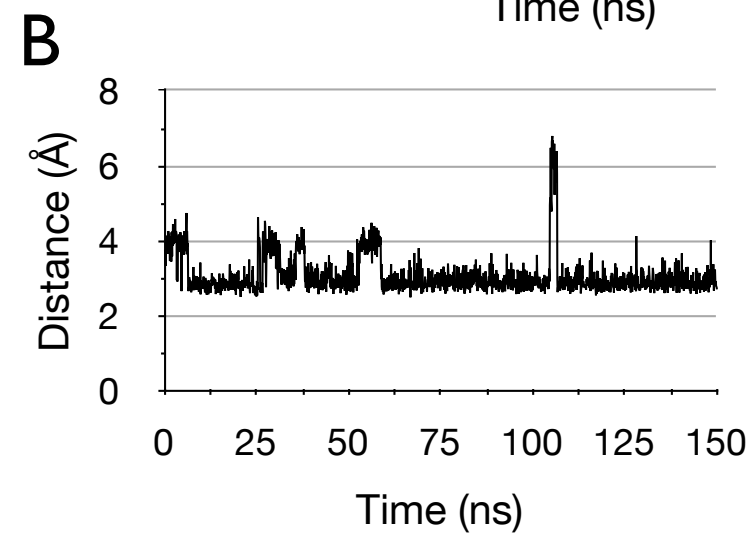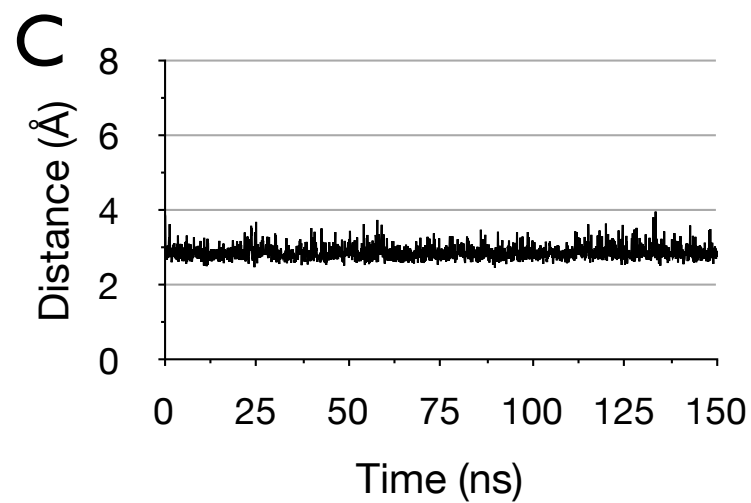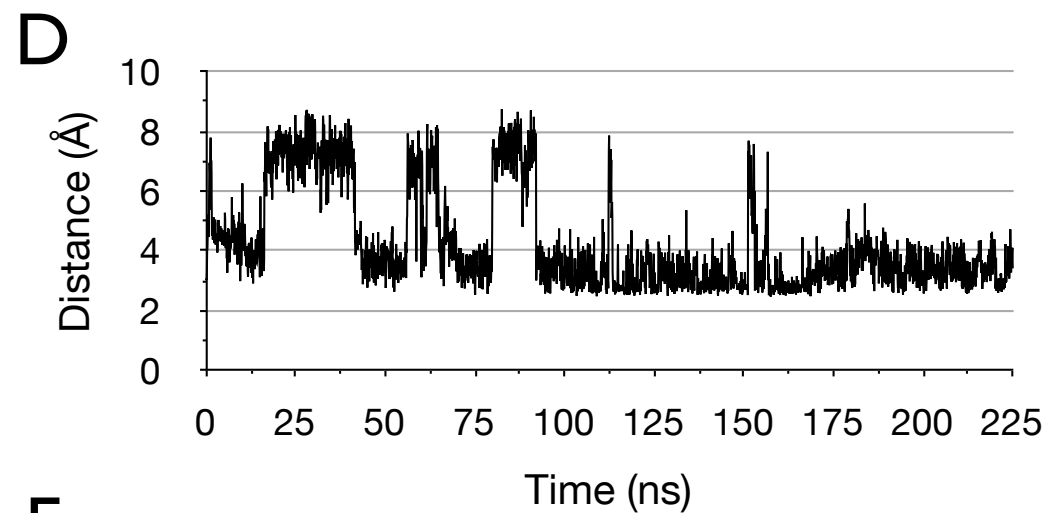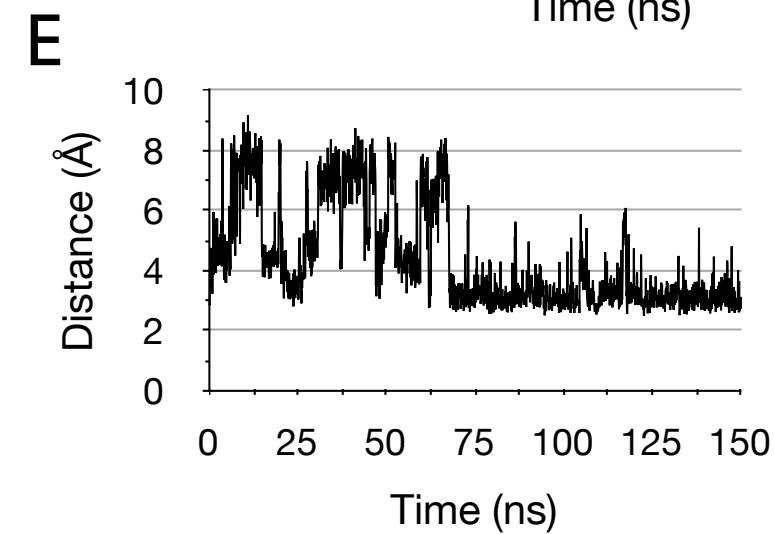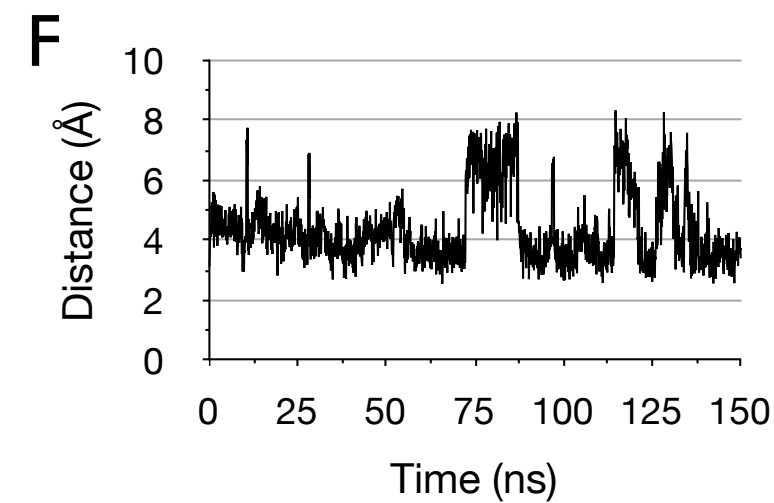

Supplement: Figure S3 — Interaction of the C-motif with the nucleotide in the ADP+Pi/apo simulations. (A-C) Time series of the distance between the C-motif serine hydroxyl oxygen and the proximal nucleotide β-phosphate oxygen for ADPPi runs 1–3 respectively. (D-F) Time series of the minimum distance between either the oxygen or nitrogen of the C-motif glutamine (LSGGQ) sidechain amido, and the proximal nucleotide ribose hydroxyl oxygen, for ADPPi runs 1–3 respectively. (PDF) [file pone.0059854.s003.pdf]
